# Partial dynamical symmetry versus quasi dynamical symmetry examination within a quantum chaos analyses of spectral data for even–even nuclei

**DOI:** 10.1038/s41598-021-95847-1

**Published:** 2021-08-12

**Authors:** H. Sabri, S. K. Mousavi Mobarakeh, A. J. Majarshin, Yan-An Luo, Feng Pan

**Affiliations:** 1 Department of Physics, University of Tabriz, 51664 Tabriz, Iran; 2 School of Physics, Nankai University, Tianjin, 300071 China; 3 Department of Physics, Liaoning Normal University, Dalian, 116029 China; 4 Department of Physics and Astronomy, Louisiana State University, Baton Rouge, LA 70803-4001 USA

**Keywords:** Theoretical nuclear physics, Statistical physics

## Abstract

Statistical analyses of the spectral distributions of rotational bands in 51 deformed prolate even–even nuclei in the 152 ≤ A ≤ 250 mass region $R_{{4_{1}^{ + } /2_{1}^{ + } }} \ge 3.00$ are examined in terms of nearest neighbor spacing distributions. Specifically, the focus is on data for 0^+^, 2^+^, and 4^+^ energy levels of the ground, gamma, and beta bands. The chaotic behavior of the gamma band, especially the position of the $2_{\gamma }^{ + }$ band-head compared to other levels and bands, is clear. The levels are analyzed within the framework of two models, namely, a SU(3)-partial dynamical symmetry Hamiltonian and a SU(3) two-coupled quasi-dynamical symmetry Hamiltonian, with results that are further analyzed using random matrix theory. The partial and quasi dynamics both yield outcomes that are in reasonable agreement with the known experimental results. However, due to the degeneracy of the beta and gamma bands within the simplest SU(3) picture, the theory cannot be used to describe the fluctuation properties of excited bands. By changing relative weights of the different terms in the partial and quasi dynamical Hamiltonians, results are obtained that show more GOE-like statistics in the partial dynamical formalism as the strength of the pairing term is increased. Also, in the quasi-dynamical symmetry limit, more correlations are found because of the stronger couplings.

## Introduction

The existence of rotational bands in atomic nuclei is a clear signature of the dominance of quadrupole deformation with axial symmetry. These deformations are caused by long-range correlations between the valence nucleons, especially when the proton and/or neutron shells are filled partly. The rotational symmetry is a clear indication of the presence dynamical symmetries^1–9^. Different approaches based on the Bohr- Mottelson geometric collective model (BMM) or interacting boson model (IBM) were applied to resolve these degeneracies between beta ($\beta$) and gamma (*γ*) rotational bands. Partial dynamical (PD) and quasi dynamical (QD) symmetries are the most commonly used methods in which their advantages are expressed in Refs.^10–20^.

Random matrix theory is a powerful technique in the investigation of nuclear structure, emphasizing the statistical properties of energy spectra. Different nuclei's statistical properties have been studied in another type of literature and get some obvious dependence of chaotic properties to nuclear structure properties such as mass, spin, and dynamical symmetries^21–30^. Rotational bands are not the subject of individual consideration, and we can suggest some references, such as^27–36^, which compare the spectral statistics of such states versus deformed ones. On the other hand, Guhr et al. have stated in their paper^37^, that degeneracy makes irregular statistics in the energy spectra of atomic nuclei. Partial dynamical symmetries (PDS)^18–20^ and quasi dynamical symmetry (QDS)^5^ are the most popular frameworks which are used to consider the properties of rotational bands.

In this study, we focused on the spectral statistics of the first three bands of deformed nuclei, namely ground, beta, and gamma bands. Experimental data^38^ and the results of both PDS and QDS formalisms are used in this research to get some obvious relation between the properties of levels and fluctuation properties.

## Theoretical frameworks

Rotational bands are the subject of the majority of recent studies about nuclear structure. Degeneracies in energy spectra are one appearance of the dynamical symmetries (DSs) underlying nuclear structure's collective properties. These collectivities were investigated via different models in which the interacting boson model (IBM) and other extended of it regarded as the most commonly used ones. These models describe various properties of nuclear structures by using the Casimir operators of dynamical symmetry groups. In the IBM framework, the SU(3) dynamical symmetry represents axially deformed nuclei corresponding to the chain U(6) ⊃ SU(3) ⊃ O(3)^1^.

The basis states in this limit labeled in the $\left| {N,\left( {\lambda ,\mu } \right),L,K} \right\rangle$ format which the N is the total number of bosons, (*λ*, *μ*) presents the irreducible representation (irreps) of SU(3) dynamical symmetry, *L* regards the angular momentum or irreducible representation of the O(3), and *K* is multiplicity label. Different *K*-values describe rotational bands. The lowest irrep of SU(3) is (2N,0), which contains only a single sequence of states with *K* = 0. The first excited bands are *β*-vibration and γ-vibration bands, which correspond by K = 0 and 2, respectively, and labeled by (2N-2, 2). This similarity of $\left( {\lambda ,\mu } \right)$ quantum numbers makes a degeneracy for the levels that have the same L values in beta and gamma bands. In contrast, those have the same L values in beta and gamma bands, while experimental energy levels have no degeneracy. Partial dynamical^18–20,39^ and quasi dynamical^5,40–42^ symmetries are the most used techniques applied in the IBM framework to remove this degeneracy in the deformed nuclei.

### SU(3) dynamic and SU(3)-partial dynamical symmetry

In IBM, the axially deformed nuclei are classified in the SU(3)-DS, and their Hamiltonian is written as a linear combination of Casimir operator of SU(3) and O(3) groups, which make a degeneracy of beta and gamma bands. Leviatan has introduced partial dynamical symmetry by adding some terms which correspond to a particular SU(3) symmetry breaking but preserves the useful aspects of dynamical symmetry^18^. A two-body SU(3)-PDS Hamiltonian in IBM-model has formed:

1 $$\begin{aligned} \hat{H}_{PDS} = & \overset{\lower0.5em\hbox{$\smash{\scriptscriptstyle\frown}$}}{H} \left( {h_{0} ,h_{2} } \right) + \hat{C}(O(3)) = \hat{H}_{DS} + \left( {h_{0} - h_{2} } \right)P_{0}^{\dag } P_{0} \\ = & h_{0} P_{0}^{\dag } P_{0} + h_{2} P_{2}^{\dag } .\tilde{P}_{2} + \hat{C}(O(3)){,} \\ \end{aligned}$$

In this Hamiltonian,$$P_{0}^{\dag } = d^{\dag } .d^{\dag } - 2\left( {s^{\dag } } \right)^{2}$$ and $$P_{2\mu }^{\dag } = 2d_{\mu }^{\dag } s^{\dag } - \sqrt 7 \left( {d^{\dag } d^{\dag } } \right)_{\mu }^{(2)}$$ describe the boson pair operator with L = 0 and 2 angular momentum, respectively, and *h*_0_ and *h*_2_ coefficients describe their effects. Also,$$\hat{C}(O(3))$$ presents the Casimir operator of the O(3) dynamical group. For *h*_0_ = *h*_2_, the Hamiltonian involves the Casimir operators of the algebras in the chain U(6) ⊃ SU(3) ⊃ SO(3), hence exhibits an SU(3) DS. Also, for *h*_0_ ≠ *h*_2_, the SU(3) symmetry is broken. In the *h*_0_ = *h*_2_ case, $$\overset{\lower0.5em\hbox{$\smash{\scriptscriptstyle\frown}$}}{H} \left( {h_{0} ,h_{2} } \right)$$ is equal with an SU(3) scalar and for *h*_0_ = −5*h*_2_ case, $$\overset{\lower0.5em\hbox{$\smash{\scriptscriptstyle\frown}$}}{H} \left( {h_{0} ,h_{2} } \right)$$ transforms as $$\left( {\lambda ,\mu } \right)$$ = (2,2), SU(3) tensor component. The solvable states of ground and gamma bands and consequently, the energy spectra of the different levels in these bands introduced by Leviatan as following^18^:

2a $$\left[ {g,K = 0} \right], \, \left| {N,\left( {2N,0} \right),K = 0,L} \right\rangle {, }E_{PDS} = CL\left( {L + 1} \right), \, L = 0,2, \ldots ,2N$$

2b $$\begin{gathered} \left[ {\gamma^{K} ,K = 2k} \right], \, \left| {N,\left( {2N - 4k,2k} \right),K = 2k,L} \right\rangle , \, \hfill \\ E_{PDS} = 6h_{2} k\left( {2N - 2k + 1} \right) + DL\left( {L + 1} \right), \, L = K,K + 1, \ldots ,(2N - 2k) \hfill \\ \end{gathered}$$

and finally, the energy spectra of the beta band are defined as:

2c $$E_{\beta } = 4N\left( {2h_{0} + h_{2} } \right) + DL\left( {L + 1} \right){\text{, For large }}N$$

The two parameters, D and *h*_2,_ are determined compared to experimental data, which detailed about such processes are available in the Refs.^19,20^. Also, the *h*_0_ parameter was varied so as to reproduce the band-head energy of the *β* band. The values of the Hamiltonian parameters derived microscopically from various EDFs, are given in^12^. For SU(3)-PDS, *h*_0_/*h*_2_ = 2, while in most self-consistent mean-field calculations, 1.9 < *h*_0_/*h*_2_ < 2.8, consistent with values obtained in global IBM fits in the rare-earth region^15^. By adding this term, the beta-gamma degeneracy is breaking, and results expressed good conformity with experimental energy levels. Even in some nuclei movement in beta and gamma experimental energy levels is also observed in energy levels while the PDS formalism can describe such nuclei correctly.

### SU(3)-quasi dynamical symmetry

The quasi dynamical symmetry is considered a powerful framework to study the symmetries associated with the two phases that appear to persist despite relatively strong symmetry-breaking interactions^5^, similar to what happened in the rotational bands of axially deformed prolate nuclei.

Rowe expressed in his papers^41^ that the quasi dynamical symmetry provides us a possibility to express a subset of physical data for systems with symmetry, which, in fact, does not have. Suppose the experimental data obviously shows that a significant subset of observed data exhibits all the properties of symmetry. In that case, this perhaps demonstrates but more detailed data on different observables reveal the symmetry broken.

To consider the rotational bands of axially deformed nuclei, we have used the Hamiltonian introduced by Thiamova et al. in Ref.^5^ which constructed of two coupled-SU(3) rotors, belonging to irreps (*λ*_1_*,* 0) and (*λ*_2_*,* 0), respectively, in the following form:

3 $$\hat{H} = A_{1} L_{1}^{2} + A_{2} L_{2}^{2} - \frac{1}{2}\chi Q_{1} \cdot Q_{2} {,}$$

1 and 2 indices denote the (*λ*_1_*,* 0) and (*λ*_2_*,* 0) irreps, and Q describes the quadrupole interaction. Pure SU(3) bands are proportional by the strong-coupling limit. This means states have good coupled-SU(3) quantum numbers, which this requirement yield via *A*_1_ = *A*_2_ = 3 and *χ* = 4. If we decrease the *χ* values*,* states show mixtures of the strongly-coupled irreps. which such phenomena are reported for different nuclei. When one analyzes the properties of the spectra yield by using different values of *χ* and the SU(3) composition of mixed states (corresponding to *χ* ≠ 4), it's apparent that SU(3) is a remarkably good dynamical symmetry for *χ* ≥ 1 and for 0*.*5 ≤ *χ* ≤ 1 condition, it regards as a good quasi-dynamical symmetry.

### Statistical analyses by RMT

RMT and its different statistics are used to connect the statistical properties of energy spectra and quantum chaos. The Nearest Neighbor Spacing Distribution (NNSD) is the most commonly used statistics for describing the statistical situation compared to different limits of RMT. A complete and pure level scheme is necessary for such analyses. A limited number of nuclei can satisfy such requirements. Therefore, a combination of different level schemes must happen. Also, to get a sequence of unit mean level spacing, we must unfold sequences, in which we followed the unfolding procedure given in Ref.^25^. Suppose we have a sequence of energy levels $E_{1} \le E_{2} \le ... \le E$. The integrated (or cumulative) level density is defined as follows,

4a $$N(E) = \sum\limits_{i = 1}^{n} {\Theta (E - E_{i} ),}$$

where $\Theta (E)$ is the Heaviside step function. The function $N(E)$ can be decomposed into two parts, a smooth average part, and a fluctuation part

4b $$N(E) = N_{av} (E) + N_{fluct} (E),$$

The fluctuation part is used to compare different systems that may have different average behavior. Consequently, in practice, one carries out the unfolding process to get rid of the average smooth part. Technically speaking, one performs a mapping from the old variables $E_{i}$ to the new variable $\varepsilon_{i}$ with $\varepsilon_{i} = N(E_{i} )$. In other words, the integrated level density is a straight line in the new variables. The mean spacing is a constant, scaled to unity. The unfolding procedure is by no means unique as it depends on the way the decomposition (4b) is performed.

We fix the $N_{av} (E_{i} )$ by taking a smooth polynomial function of degree 6 to fit the staircase function $N(E)$. We obtain finally, the unfolded spectrum with the mapping

4c $$\{ \varepsilon_{i} \} = N(E_{i} ),$$

The nearest neighbor level spacing is defined as $s_{i} = (\varepsilon_{i + 1} ) - (\varepsilon_{i} )$. The distribution $P(s)$ presents the probability of $s_{i}$ to lie within the infinitesimal interval $[s,s + ds]$. For nuclear systems with time-reversal symmetry in which spectral spacing follows Gaussian Orthogonal Ensemble (GOE) statistics, the NNS probability distribution function is well approximated by Wigner distribution:

5a $$P(s) = \frac{1}{2}\pi se^{{ - \, \frac{{\pi s^{2} }}{4}}} {,}$$

which have been used to exhibit the chaotic properties of considered spectra. On the other hand, the fluctuation properties of non-chaotic systems, i.e., regular systems, follow the Poisson distribution:

$$P(s) = e^{ - s} {,}$$

Different studies on physical systems^43–61^ showed that the NNS distributions are located between Poisson and chaotic (GOE) limits. This forces us to employ distribution functions that compare the spectral statistics of considered systems with both regular and chaotic limits quantitatively. The Abul-Magd distribution is one of the popular distributions and we are supposed to note that the energy level spectrum is a product of the superposition of independent subspectra. This distribution is based on the Rosenzweig and Porter random matrix model^25^. The straightforward form of this distribution proposed by Abul-Magd et al*.* as:

6 $$P(s,q) = \left[ {1 - q + q(0.7 + 0.3q)\frac{\pi s}{2}} \right] \times \exp \left( { - (1 - q)s - q(0.7 + 0.3q)\frac{{\pi s^{2} }}{4}} \right),$$

in which a quantitative measure interpolates between Poisson (*q* = 0) and GOE statistics (*q* = 1). We used the maximum-likelihood (ML) method to determine the best-fit Abul-Magd parameter, *q*. This method is completely independent from the binning procedure, since it is directly applied to the raw data in contrast to a least-squares-fitting approach. The uncertainty of *q* is conservatively approximated by the half-width at half maximum (HWHM) of the likelihood distribution^30^.

## Results

We present our results in two separate sections. In the first part, the experimental data of 0^+^, 2^+^, and 4^+^ levels in the ground, gamma, and beta bands are analyzed to get signatures of correlation between these levels in considered nuclei. In the second part, we studied the fluctuation properties of energy spectra, which yield via Eqs. (1, 3) for PDS and QDS formalisms. To get a meaningful description of rotational bands' statistical situation, we analyzed sequences with at least 25 spacing. Then, the qualified sequences unfolded and studied by the Abul-Magd distribution and MLE technique. The degree of chaotic dynamics for each sequence measure by the Abul-Magd distribution's parameter, i.e., *q*. On the other hand, the majority of most short sequences' exploration makes an overestimation of this quantity. Therefore, we would not concentrate only on these quantities' implicit values and compare their values in different sequences.

### Statistical analyses by using experimental data

We used all the available empirical data^38^ for deformed eve-even prolate nuclei to get a relevant result. 0^+^, 2^+^, and 4^+^ levels are selected due to their relative abundance in such nuclei. These nuclei have an energy ratio of $R_{{{{4_{1}^{ + } } \mathord{\left/ {\vphantom {{4_{1}^{ + } } {2_{1}^{ + } }}} \right. \kern-\nulldelimiterspace} {2_{1}^{ + } }}}}$ > 3.00 and located in the 152 ≤ A ≤ 250 as listed in Ref.^20^. We used the explicit values of energy levels and ignored the uncertainties due to the experimental methods for their determination. Levels are classified as different bands and also their spin values. The results are shown in Table 1.

**Table 1:** *q* values, chaoticity parameter, for different levels of rotational bands determined via Eq. (6) and experimental data.

Level	*N*	*q*
All levels in ground band	102	0.29 ± 0.05
All levels in beta band	148	0.19 ± 0.03
All levels in gamma band	102	0.38 ± 0.08
$2_{g}^{ + }$	51	0.62 ± 0.04
$4_{g}^{ + }$	51	0.59 ± 0.07
$0_{\beta }^{ + }$	51	0.58 ± 0.09
$2_{\beta }^{ + }$	51	0.41 ± 0.12
$4_{\beta }^{ + }$	46	0.29 ± 0.05
$2_{\gamma }^{ + }$	51	0.87 ± 0.10
$4_{\gamma }^{ + }$	51	0.79 ± 0.11

*N* is the number of spacing in each sequence, which is from 51 nuclei. Sequences are constructed of experimental data.

Results show a significant difference between the spectral situations of these sequences, where the correlation between the levels of gamma-band is obvious. In all bands, levels with low spin show more GOE-like statistics. Also, the beta vibrations affect the correlation of levels, and different levels of this band, especially the state, deflect from the GOE limit. On the other hand, gamma vibrations keep the correlation of levels. These results may be interpreted as the difference between vibration on radius and angles, making different changes in the shape of nuclei.

#### Statistical analyses by using theoretical predictions of PDS formalism

In the following parts of the paper, we focused on the theoretical predictions for considered levels by using different formalisms. As have stated in detail in Refs.^18,20^, the SU(3) dynamical symmetry makes a degeneracy of levels having the same spin of the *β*- and *γ*-bands of the lowest excitation energy $K \, = \, 0^{ + }$ and $K \, = \, 2^{ + }$ irreducible representations $(\lambda ,\mu ) = (2N - 4,2)$, respectively^2^. The experimental spectrum of deformed nuclei and especially the $\beta \left( {K = 0_{2}^{ + } } \right)$ and $\gamma \left( {K = 2_{1}^{ + } } \right)$ bands are not degenerate, and our results in Table 1, which suggest different statistical behavior, confirm this result. This means the spectrum of an exact SU(3)-DS obtained by *h*_0_ = *h*_2,_ deviates considerably from the empirical data. On the other hand, the abilities of PDS to reproduce experimental data in deformed prolate nuclei are shown in Refs.^18,20^. In PDS formalism, by adding a term to Hamiltonian of SU(3) dynamical symmetry, the *β*–*γ* degeneracy is broken, and theoretical energy levels have good conformity with experimental levels. To compare the accuracy of these two formalisms in comparison of the two formalism compared with the results of Table 1, we determined the chaoticity parameter of considered levels via these models' predictions. To this aim, we determined the constants of Eqs. (2a–2c) by the method introduced by Leviatan in Ref.^18^. The *C* and *h*_*2*_ parameters are determined as the experimental energy difference in specific levels, namely $C = \left[ {E\left( {2_{g}^{ + } } \right) - E\left( {0_{g}^{ + } } \right)} \right]$ and $h_{2} = \left[ {E\left( {2_{\gamma }^{ + } } \right) - E\left( {2_{g}^{ + } } \right)} \right]$. Also, the *h*_*0*_ was varied in order to reproduce the headband energy of the *β* band. The statistical analysis results with both DS and PDS formalisms are presented in Table 2.

**Table 2:** Similar to Table 1, *q* values describe the statistical situation of considered levels determined by DS and PDS formalism.

Level	*N*	*qDS*	*qPDS*
All levels in ground band	102	0.31 ± 0.06	0.30 ± 0.05
All levels in beta band	148	0.28 ± 0.10	0.21 ± 0.04
All levels in gamma band	102	0.45 ± 0.11	0.39 ± 0.06
$2_{g}^{ + }$	51	0.64 ± 0.03	0.65 ± 0.03
$4_{g}^{ + }$	51	0.62 ± 0.04	0.61 ± 0.02
$0_{\beta }^{ + }$	51	0.67 ± 0.08	0.64 ± 0.03
$2_{\beta }^{ + }$	51	0.57 ± 0.14	0.45 ± 0.05
$4_{\beta }^{ + }$	46	0.47 ± 0.15	0.38 ± 0.03
$2_{\gamma }^{ + }$	51	0.57 ± 0.18	0.84 ± 0.07
$4_{\gamma }^{ + }$	51	0.47 ± 0.14	0.75 ± 0.03

Only for $2_{g}^{ + }$ level, the results of DS formalism are closer to experimental data, and for other levels, the PDS predictions are in satisfactory agreement with the results reported in Table 1. Furthermore, we got a similar tendency in spectral analyses by using the PDS predictions, for example, the more correlation in the levels of gamma-band and low spin states. This will consider in the next section of the paper. The great deviations of DS results for the levels of beta and gamma bands confirm PDS formalism's advantages in describing rotational bands. The effect of conservation of K quantum number makes a GOE-like behavior in pure sequences which contain levels with single values of both J and K quantum numbers. On the other hand, the broken K values which yield in mixed sequences, categories of levels with a single value of J, and several possible values of K, show deviation of GOE limit and make Poisson-like statistics. Also, the breaking of K-symmetry in the considered nuclei are strong in the gamma band and it makes more regular of the levels of the beta band in comparison with other rotational bands.

**Figure 1:**
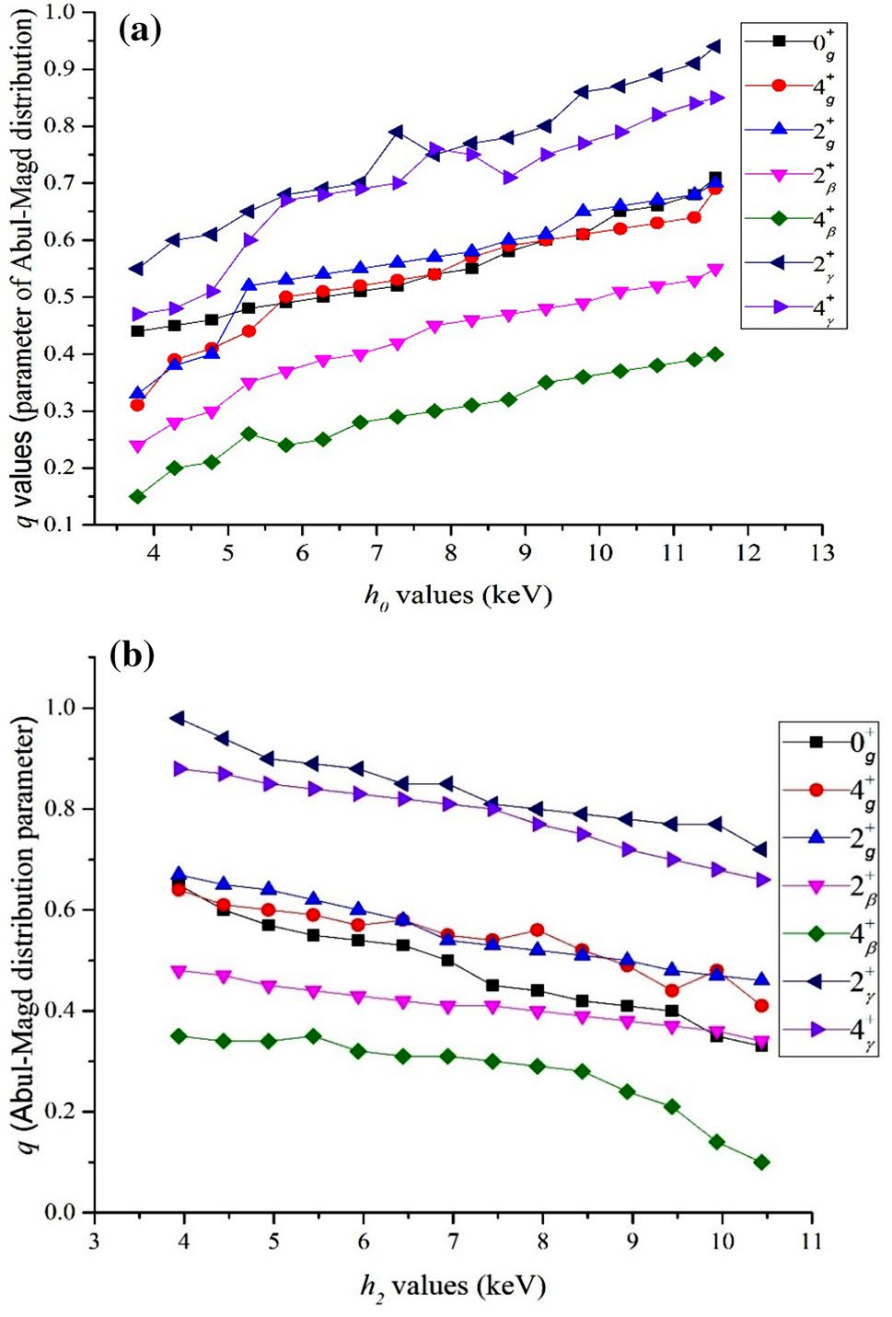
The variation of chaoticity degree, *q* values, for energy levels of considered nuclei determined by using (**a**) different *h*_*0*_ (**b**) different *h*_*2*_ values in the PDS framework.

In Ref.^20^, it has been shown the feasibility of PDS formalism in determining different energy levels of different prolate deformed nuclei in detail. The results confirm the advantages of PDS compared to the DS framework in the reproduction of energy levels without any degeneracy of beta and gamma bands. In this manner, the values of *h*_*0*_ and *h*_*2*_ parameters are yield in the [3.78–11.56] and [3.94–10.77] intervals, respectively (all in keV). These quantities describe the effect of scalar and quadrupole parts of Hamiltonian on the energy spectra. In this part, we look at the effect of different parts of the Hamiltonian by using spectral statistics. We determined the different energy levels by using the *h*_0_ coefficients, which varied in the specific interval with Δ*h*_0_ = 0.5 keV step changes while the *h*_2_ assumed to be 5.89 keV, the averaged value of this quantity for the considered nuclei. We then repeat this process by using different *h*_*2*_ values, which varied in their interval with Δ*h*_2_ = 0.5 keV step changes and assumed *h*_0_ = 6.45 keV. Results are presented in Fig. 1a,b for the variation of *h*_*0*_ and *h*_*2*_, respectively.

PDS results show the same tendency in the spectral statistics of different levels and suggest the more chaotic behavior in all sets of *h*_0_ and *h*_2_ coefficients, similar to what has been reported using the experimental data. Additionally, more GOE-like behaviors are yield via the maximum value of *h*_*0*_ and minimum values of *h*_*2*_. One may conclude the correlation of the levels is the result of the pairing effect on the energy spectra while, the Poisson-like behavior in the spectral statistics yield, is due to the quadrupole interaction.

#### Statistical analyses by using theoretical predictions of QDS formalism

**Figure 2:**
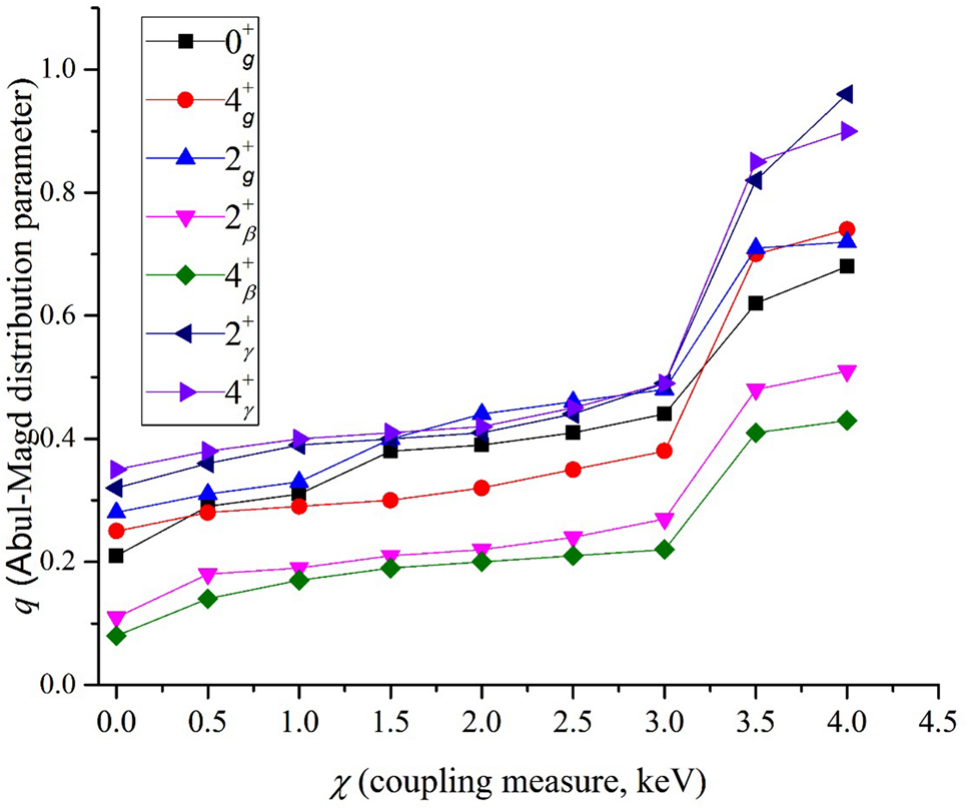
The variation of chaoticity degree, *q* values, for different energy levels determined by using different *χ* values in the QDS framework, Eq. (3), and A_1_ = A_2_ = 3.

This part's results are independent of any special nuclei and describe the relationship between coupling and fluctuation properties. Similar to what we have done by PDS formalism, we focused on Eq. (3) and determined different energy levels as the expectation value of Hamiltonian QDS formalism in different coupling conditions defined as the *χ* values. To this aim, we have followed the method explained by Thiamov et al. in Ref.^5^ and used the same quantum number to describe eigen-states as the PDS part to evaluate the energy spectra. They have supposed $\lambda_{1} = 14$ and $\lambda_{2} = 8$ to get enough large number of each level for their analyses. We supposed $\lambda_{1} = 8$ and $\lambda_{2} = 5$ which can provide our considered 7 levels which we focused on their spectral fluctuation. The strong coupling is yield by A_1_ = A_2_ = 3 and *χ* = 4 (all in keV) requirement, and we supposed the same amounts for A_1_ and A_2_ coefficients. On the other hand, we determined energy levels using different χ values that varied in the [0–4] by Δ *χ* = 0.5 keV step changes. The results are reported in Fig. 2.

The results of QDS formalism for the spectral statistics of different levels agree with the results yield by using experimental data where the most correlation suggested at the $2_{\gamma }^{ + }$ level. On the other hand, for weak coupling, the results suggest Poisson-like statistics for all levels, and when the coupling reaches the maximum value, the spectral statistics of all levels show GOE-like behavior. The apparent regularity for weak coupling confirms the GOE limit predictions, suggesting more regular dynamics for deformed nuclei than the spherical nuclei (e.g., magic or semi-magic nuclei have strong coupling). One can expect the spherical nuclei, which have shell model spectra shown predominantly less regular dynamics in comparison with the deformed ones. This result is known as the AbulMagd-Weidenmuller chaoticity effect^53,54^ which suggests the suppression of chaotic dynamics due to the rotation of nuclei. These results for the chaotic behavior of two-rotor models suggest a GOE-like behavior for solvable coupled systems. The spectrum of such systems is simply the combination of the SU(3) and O(3) Casimir operators, and hence the system is still within the SU(3) limit. On the other hand, as have reported in Ref.^62^, different separable potentials for simple two-dimensional oscillators present chaotic behavior. They concluded that momentum coupling is more fundamental to the chaoticity of systems than metric coupling. We suggested the same conclusion as shown in the suppression of chaotic dynamics.

**Figure 3:**
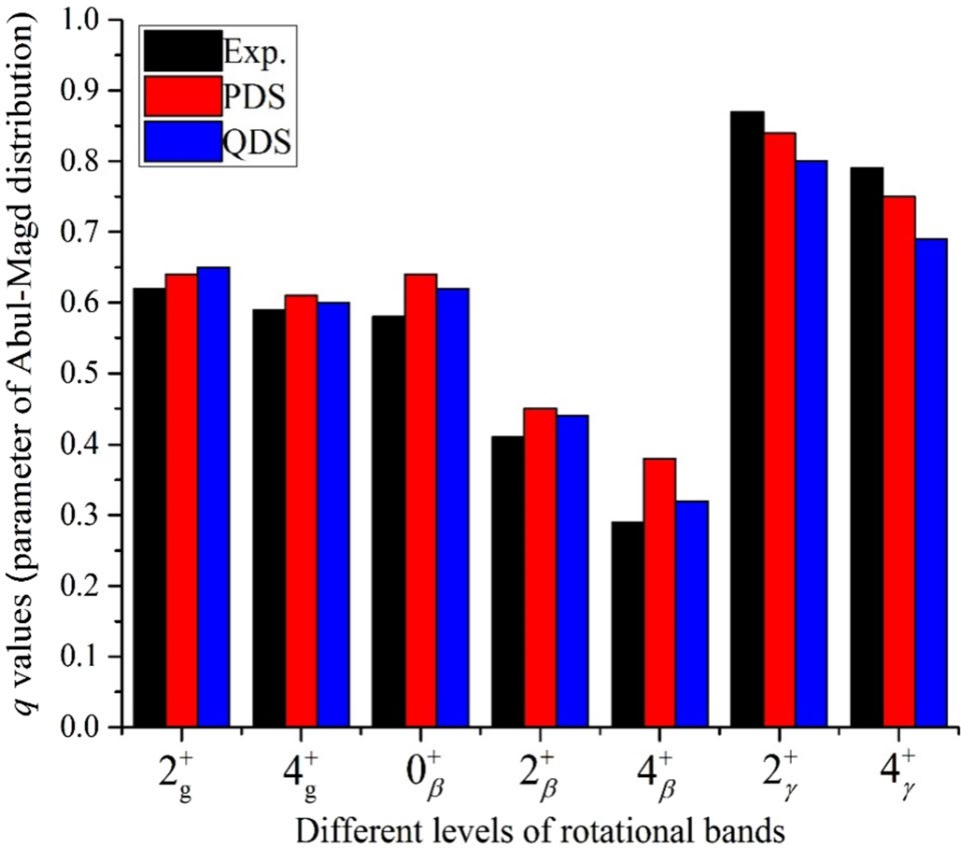
The chaoticity measure of different levels yields by using the experimental data and PDS and QDS results.

A comparison between the statistical analyses of the different energy levels that are results of (a) experimental data, (b) PDS, and (c) QDS formalisms are presented in Fig. 3. The results show the advantages of both models, which suggest results with a 5% difference in the *q* values.

To get the results of QDS formalism for considered nuclei, we consider *A*_1_ = *A*_2_ = 3 and the average values of *χ* quantities in the Eq. (3), which describe the coupling amounts for all the levels of rotational bands in the considered nuclei are yield as 2.94, 3.31 and 2.87 for ground, gamma and beta bands, respectively. The results driven of Fig. 2 explain the most chaotic behavior of the gamma band in comparison with two other rotational bands. Also, the difference in this quantity for ground and beta bands describes the partly regular behavior of these bands and Poisson-like behavior of levels in the beta band due to weak coupling. In the ground band, both theoretical methods have the same efficiency and make the same results, but in the beta band, the results of QDS are in better agreement with experimental results. On the other hand, in the gamma band, we got better accommodations by PDS formalism results. One may conclude that PDS is a successful method for such structures with small deformation, which reserve the system's symmetries. Also, in all rotational bands, QDS formalism has more accurate results than the PDS technique when the spin of states increased. In Refs.^63–67^, Macek et al., suggested the rotational bands as the results of an adiabatic separation of collective rotations built upon a subset of intrinsic vibrational states IBM framework. They offer regularity to intrinsic vibrational mode versus chaoticity, which yields due to collective rotations. With our predictions about the more accuracy of QDS in the rotational bands, these results may suggest the application of this model in the investigation of high spin states. We will consider this in the following studies. We tried to consider a similar investigation by using the levels of the single nucleus to consider the properties of different Hamiltonians. The lack of enough data for an acceptable statistical analysis, at least 25 samples, force us to consider the experimental data which are collected of different nuclei.

## Summary and conclusion

We have seen the spectral statistics of different levels of rotational bands in deformed prolate even–even nuclei of both PDS and QDS formalisms in the framework of NNSD statistics of RMT. The obvious differences between the chaoticity degrees of beta and gamma bands suggest different vibrations on the level correlation. We have compared the NNSD of PDS and QDS results. We have achieved GOE-like statistics for PDS formalism when the effect of pairing terms increased. Likewise, we found more correlation for the strong coupling in the QDS formalism. The variation of the pairing and quadrupole terms in the PDS and different coupling in the QDS formalisms and the effect of these changes on the spectral statistics are significant and suggest detailed analyses about them in future studies.
